# Comparative Analysis Using Pulsed-Field Gel Electrophoresis Highlights a Potential Transmission of *Salmonella* Between Asymptomatic Buffaloes and Pigs in a Single Farm

**DOI:** 10.3389/fvets.2020.552413

**Published:** 2020-11-10

**Authors:** André Marcos Santana, Daniela Gomes da Silva, Renato Pariz Maluta, Lucas José Luduverio Pizauro, Kalina Maria de Medeiros Gomes Simplício, Clarissa Helena Santana, Sarah de Andrade Dias Rodrigues, Dália dos Prazeres Rodrigues, José Jurandir Fagliari

**Affiliations:** ^1^Department of Veterinary Medicine, Maringá State University (UEM), Maringá, Brazil; ^2^Department of Veterinary Clinic and Surgery, School of Agricultural and Veterinary Sciences, São Paulo State University (FCAV/UNESP), São Paulo, Brazil; ^3^Department of Veterinary Pathology, School of Agricultural and Veterinary Sciences, São Paulo State University (FCAV/UNESP), São Paulo, Brazil; ^4^National Reference Laboratory Diagnosis of Enteric Bacteria, Oswaldo Cruz Institute, Oswaldo Cruz Foundation (FIOCRUZ), Rio de Janeiro, Brazil

**Keywords:** *Bubalus bubalis*, epidemiology, feces, Jafarabadi, porcine, *Salmonella* Agona, serotypes, swines

## Abstract

Buffaloes and pigs play an important epidemiological roll in the *Salmonella* infection cycle, and asymptomatic animals can act as key component in the dissemination of the disease by horizontal, vertical, and cross-species transmission. Our study aimed and was able to confirm evidences of a cross-species transmission of *Salmonella* Agona between asymptomatic buffaloes and pigs. Also, we described *Salmonella* infection within the pig production phases, involving serotypes Agona, Senftenberg and Schwarzengrund. Rectal samples were collected from Jafarabadi buffaloes (*n* = 25) and Piau pigs (*n* = 32), located on a single farm. *Salmonella* Agona was isolated from lactating buffaloes, gilts, pregnant sows, and weaned pigs, *Salmonella* Schwarzengrund from lactating sows and *Salmonella* Senftenberg from gilts, pregnant sows, lactating sows, and weaned pigs. Pulsed-field Gel Electrophoresis protocol (PFGE) was performed and revealed four different profiles. Profile 1 (*Salmonella* Agona), isolated from a pregnant sow, a gilt and two lactating buffaloes, revealed a indistinguishable PFGE pattern, confirming evidences of potential cross-species transmission. Profile 2 (*Salmonella* Agona), 3 (*Salmonella* Senftenberg), and 4 (*Salmonella* Schwarzengrund), isolated from pigs, revealed important indistinguishable PFGE patterns, evidencing *Salmonella* infection within the pig production phases. Considering the epidemiological relevance of buffaloes and pigs in the cycle of *Salmonella* infection, confirmation of a potential cross-species transmission of *Salmonella* Agona and potential *Salmonella* infection within the pig production phases highlights the importance of the correct establishment of preventive health strategies in farms, in special the importance of avoiding contact between buffaloes and pigs, since cross-species transmission can occur, increasing the risk of spreading the disease.

## Introduction

*Salmonella* is one of the most important pathogens in livestock animals, and is a matter of concern, as it may be responsible for great economic losses within the herd, as well as being a zoonotic agent linked to foodborne illness and therefore a major public health concern worldwide (1). In buffaloes, *Salmonella* is also responsible for economic losses (2), and several serotypes have been reported worldwide (2–8), isolated in feces (2, 3, 5, 6), raw meat (4, 8) and milk (7). Although reports show that salmonellosis in buffalo-calves is a widespread disease characterized by gastrointestinal lesions, diarrhea, hyperthermia, and dehydration (2, 9, 10), the major source of infection in the herd are asymptomatic adult animals shedding the bacteria through the feces (2), which is a great concern since these animals can act as reservoirs and be a great risk of transmission to humans and other animal species (3).

In pigs, *Salmonella* is also a matter of concern, being that a great diversity of serotypes have been reported, isolated in pig feces, lymph nodes/tonsils, and subproducts (1, 8, 11–18). *S*. Choleraesuis, serotype adapted to pigs, often cause the septicemic form of the disease, while *S*. Typhimurium is responsible for the enterocolitic form of the disease (19, 20). On the other hand, several *Salmonella* serotypes have been linked to asymptomatic pig carriers, being the main risk factor for the contamination of the final product during harvest and thereby presenting a food safety concern (21). Therefore, asymptomatic pigs represent a constant risk of *Salmonella* transmission to humans and other animal species (1, 22). Pork meat has been considered one of the major food products of animal origin responsible for *Salmonella* transmission to humans in diverse countries, including industrialized ones (22–24), being responsible for outbreaks in humans, as described in the literature for *S*. Agona (25), *S*. Senftenberg (26), and *S*. Schwarzengrund (27).

With this study, we aimed and we were able to show evidences of a potential transmission of *Salmonella* between asymptomatic buffaloes and pigs. Also, we were able to describe a *Salmonella* infection within the pig production phases. Therefore, these evidences highlight the importance of establishing preventive health strategies, among them avoiding contact between buffaloes and pigs, since *Salmonella* is a matter of concern for these two animal species, as well as an important public health problem worldwide.

## Materials and Methods

### The Population Studied and Number of Samples Collected

This research was approved by the Ethics Committee on Animal Use of “Faculdade de Ciências Agrárias e Veterinárias, UNESP” (Protocol no 010885-08).

Samples were collected from Jafarabadi lactating buffaloes and five categories of Piau pigs within the production chain inside the farm (pregnant sows, lactating sows, gilts, boars, and weaned pigs). These animals were located on a single farm, in São Paulo State, Brazil. The lactating buffaloes were raised in a semi-intensive system, with a diet based on roughage and chopped sugar cane, supplemented with protein concentrate. The animals were housed during the night and released to graze in paddocks during the day, after the morning milking was performed. The pigs were raised in a semi-intensive system, with a diet based on a ration containing corn and soybean meal, supplemented with mineral core. The pigs were housed during the night and released to graze in paddocks during the day. Although the categories of pigs should be allocated into different structures, appropriate to each stage of the production chain, this did not occur on this farm. Thus, animals from all stages shared the same installation, in an inadequate breeding system where all animals had direct contact. Also, buffaloes and pigs, although housed in separate facilities during the night, were released in the same paddocks during the day, and therefore had direct contact. While in the paddocks, pigs could not access buffaloes feeders and water system because they were placed to high for the pigs. Therefore, the pigs had access to feeders and water through an adapted creep feeding system located inside the paddocks, where the buffaloes had no access.

A total of 25 rectal swab samples from Jafarabadi lactating buffaloes and 32 rectal swab samples from Piau pigs were collected for microbiological isolation of *Salmonella*. Rectal swab samples were collected from different categories of pigs within the production chain inside the farm: pregnant sows (*n* = 5), lactating sows (*n* = 5), gilts (*n* = 8), boars (*n* = 3), and weaned pigs (*n* = 11). Samples were collected at only one timepoint and on the same day.

### Sampling and Initial Microbiological Procedures

To evaluate the presence of *Salmonella*, three fecal samples were collected from the rectum of each animal, with a cotton swab, that was immediately transferred into tubes containing 10 ml of the selective enrichment broths selenite cystine (SC), Muller-Kauffmann tetrathionate (MKT) and Rappaport-Vassiliadis (RV). All samples were then transported to the laboratory in thermal boxes containing ice (time range from 2 to 3 h), where all microbiological procedures were performed.

At the “Research Support Laboratory of the Department of Veterinary Clinic and Surgery, FCAV, UNESP, Jaboticabal Campus, Brazil,” selective enrichment broths were incubated at 37°C for 24 h. After incubation, the broths (SC, TMK, and RV) were seeded on plates containing modified-brilliant green agar and xylose lysine tergitol 4 (XLT4) agar and incubated (37°C, 24 h). From each plate, three colonies with morphologic characteristics that suggested *Salmonella* genus (28) were inoculated in tubes containing triple-sugar-iron agar (TSI) and lysine-agar (LIA) (presumptive biochemistry tests) and incubated (37°C, 24 h).

### Serotyping

After biochemical confirmation, slide agglutination tests were performed using somatic and flagellar polyvalent *Salmonella* antisera (poli-O, poli-H, and poli-D). Positive samples in slide agglutination tests were inoculated in tubes containing nutrient agar and sent to the laboratory of Enterobacteria of the Instituto Oswaldo Cruz – IOC/FIOCRUZ (Manguinhos, Rio de Janeiro, Brazil) for further serotyping.

### Pulsed-Field Gel Electrophoresis (PFGE)

Positive samples in slide agglutination tests were also inoculated in tubes containing nutrient agar and sent to the “Laboratory of Veterinary Bacteriology, FCAV, UNESP, Jaboticabal Campus, Brazil,” where they were subtyped by a standardized rapid Pulsed-field Gel Electrophoresis protocol used by laboratories in PulseNet, as described previously (29). Chromosomal DNA was digested with XbaI. Electrophoresis conditions consisted of a initial switch time of 2.2 s and a final switch time of 54.2 s (length of time the electrical field is applied on each direction) at a gradient of 6V cm^−^1 and an included angle of 120°. The gels were electrophoresed for 18 h.

### Physical Examination and Laboratory Analysis

The health status of the lactating buffaloes was verified by physical examination, performed at the same time that rectal swab samples were collected. Their feces were analyzed for signs of diarrhea, blood, and mucus. Fecal consistency scores were determined as 0, normal (firm); 1, mild diarrhea (soft); and 2, and moderate to severe diarrhea (liquid) (30). The degree of dehydration was estimated as 0, absent (normal skin turgor and bright eyes); 1, mild (skin turgor slightly decreased and eyes not retracted); and 2, moderate to severe (skin turgor decreased and eyes retracted) (30). Rectal body temperature was measured. Also, the health status was verified by hemogram interpretation, being that blood samples were collected at the same time that rectal swab samples were performed. Blood sampling was performed by puncture of the jugular vein using a vacuum collection system (25 × 8 mm needles), after local antisepsis with iodized alcohol. Blood samples were collected into siliconized plastic tubes containing EDTA (BD Vacutainer, 4.0 ml). The health status of the pigs was verified by visual inspection and by feces inspection, performed at the same time that rectal swab samples were collected.

Hemogram, including red blood cell count (RBC), hemoglobin concentration (HGB), packed cell volume (PCV), and total white blood cell count (WBC) was performed using automated hematology pocH-100iV Diff analyzer (Sysmex Corporation, Kobe, Japan). Differential WBC count was performed on blood smear stained with modified Rosenfeld dye by optical microscopy (31). Normality was evaluated by comparing the results of hemogram to those described in literature for adult lactating buffaloes (32).

## Results

### *Salmonella* Serotypes Isolated

Bacteriological isolation showed that 2 (8.0%) of the 25 rectal samples collected from Jafarabadi lactating buffaloes were positive for *S*. Agona (Table 1).

**Table 1:** Identification of *Salmonella* serotypes isolated from feces of buffaloes and pigs, as well as the profiles of *S*. Agona, *S*. Senftenberg, and *S*. Schwarzengrund identified using PFGE analysis.

**Serotypes**	**PFGE profile**	**Animal category**	**No of positive animals/no of tested animals (% of positive animals)**	**No of positive animals per serotype/no of tested animals (% of positive animals)**
*Salmonella* Agona	1	Lactating buffalo	2/25 (8.0%)	2/25 (8.0%)
		Pregnant sow	1/32 (3.13%)	3/32 (9.39%)
		Gilt	1/32 (3.13%)	
	2	Weaned pig	1/32 (3.13%)	
*Salmonella* Senftenberg	3	Pregnant sow	2/32 (6.25%)	14/32 (43.75%)
		Lactating sow	2/32 (6.25%)	
		Gilt	2/32 (6.25%)	
		Boar	2/32 (6.25%)	
		Weaned pig	6/32 (18.75%)	
*Salmonella* Schwarzengrund	4	Lactating sow	2/32 (6.25%)	2/32 (6.25%)

In pigs, 19 (59.4%) of the 32 rectal samples collected were positive for *Salmonella*. From the positive samples, 3 (15.8%) were *S*. Agona, 14 (73.7%) were *S*. Senftenberg, and 2 (10.5%) were *S*. Schwarzengrund. Additionally, *Salmonella* was detected in more than one category of a pig inside the production cycle of the farm, being positive in 3/5 pregnant sows (60%), 4/5 lactating sows (80%), 3/8 gilts (37.5%), 2/3 boars (66.7%), and 7/11 weaned pigs (63.6%) (Table 1).

### Pulsed-Field Gel Electrophoresis (PFGE) Analysis

**Figure 1:**
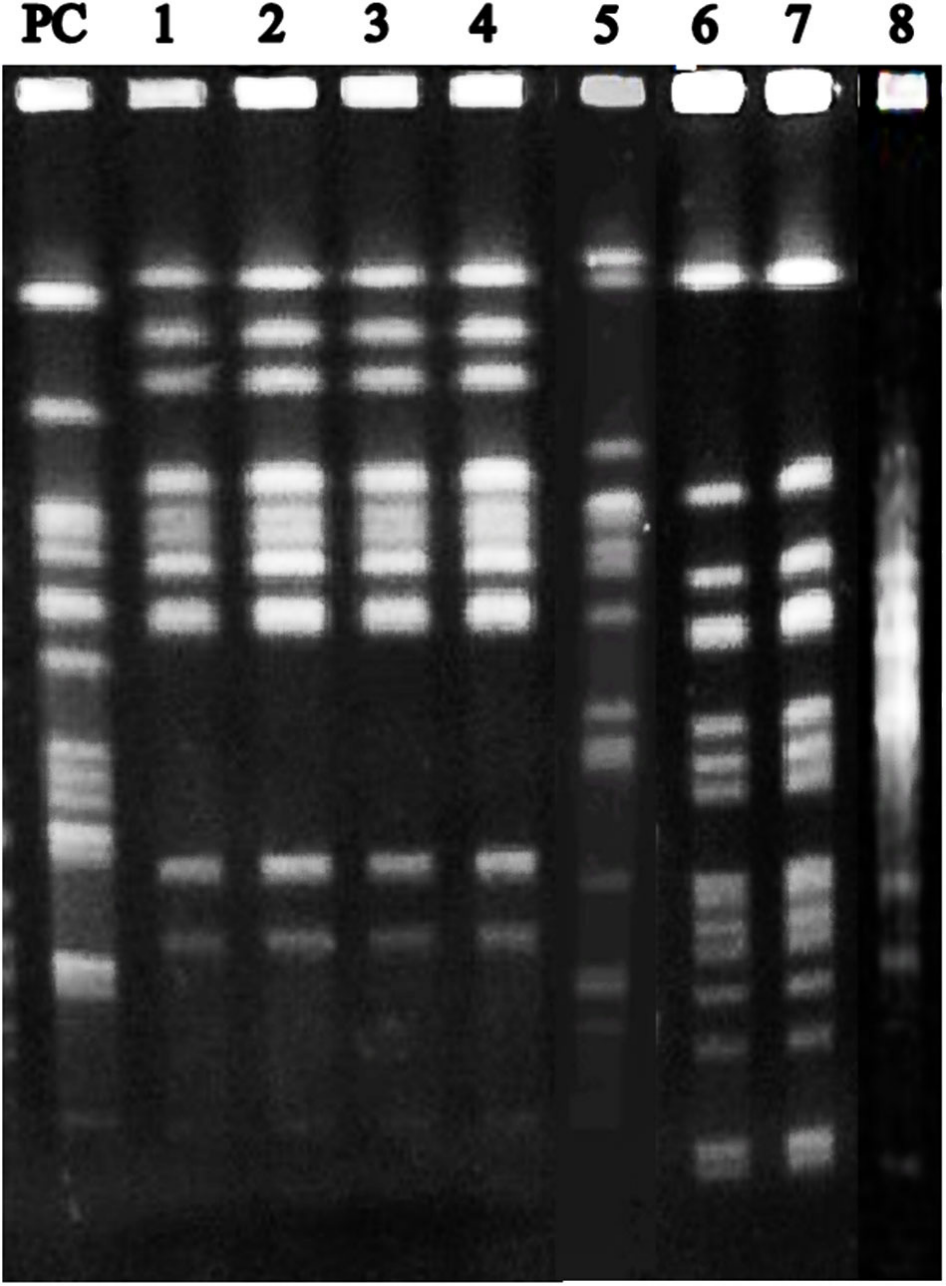
PFGE profiles of *S*. Agona, *S*. Senftenberg and *S*. Schwarzengrund isolated from feces of buffaloes and pigs. PC: positive control (pulse marker, 50–1,000 kb, Sigma-Aldrich); 1: Profile 1—*S*. Agona isolated from a lactating buffalo; 2: Profile 1—*S*. Agona isolated from a lactating buffalo; 3: Profile 1—*S*. Agona isolated from a pregnant sow; 4: Profile 1—*S*. Agona isolated from a gilt; 5: Profile 2—*S*. Agona isolated from a weaned pig; 6: Profile 3—*S*. Senftenberg isolated from a weaned pig; 7: Profile 3—*S*. Senftenberg isolated from a boar; 8: Profile 4—*S*. Schwarzengrund isolated from a lactating sow.

PFGE analysis identified two profiles (profiles 1 and 2) of *S*. Agona, one profile (profile 3) of *S*. Senftenberg and one profile (profile 4) of *S*. Schwarzengrund. Profile 1 of *S*. Agona revealed a indistinguishable PFGE pattern between the isolates identified in two categories (pregnant sow and gilt) of pigs. This same profile was also identified in the two lactating buffaloes. Profile 2 of *S*. Agona was only identified in one animal, a weaned pig (Table 1 and Figure 1).

The single profile identified for *S*. Senftenberg (profile 3) revealed a indistinguishable PFGE pattern between the isolates identified in all animal categories (sows, gilts, boars and weaned pigs) of pigs. The single profile identified for *S*. Schwarzengrund (profile 4) also revealed a indistinguishable PFGE pattern between the isolates identified in two lactating sows (Table 1 and Figure 1).

### Health Status

The health status of the lactating buffaloes was accessed by physical examination and hemogram analysis. In this sense, all buffaloes, both *Salmonella*-positive (*n* = 2) and *Salmonella*-negative (*n* = 23) animals, did not present clinical signs of salmonellosis, as well as did not present alterations in the hemogram (Table 2).

**Table 2:** Physical examination and hemogram results of the two positive *S*. Agona lactating buffaloes (*n* = 2: LB1, LB2).

		**Positive *S*. Agona LB**		**Neg.LB**	
	**Variables**	**LB1**	**LB2**	**Min–Max (*n* = 23)**	**Reference Values^a,b^**
**Physical examination**	Rectal temperature	38.1	37.9	34.2–38.3	37.5–39.2
	Feces consistency score	0	0	0	0
	Degree of dehydration	0	0	0	0
**Erythrogram**	RBC (×10^6^/μl)	6.47	6.81	5.49–8.14	4.70–10.60
	Packed cell volume (%)	29.4	34.9	27.9–38.5	26.1–45.3
	HGB (g/dl)	12.6	15.3	11.7–16.5	9.2–18.3
**Leukogram**	WBC (/μl)	9,400	14,500	7,600–11,700	5,000–14,700
	Lymphocytes (/μl)	3,196	4,470	2,464–6,120	2,400–9,100
	Segmented neutrophils(/μl)	4,794	8,689	2,754–6,555	1,800–9,100
	Band neutrophils (/μl)	0	0	0–105	0–500
	Monocytes (/μl)	0	149	0–380	0–1,200

*RBC, red blood cell count; HGB, hemoglobin concentration; MCV, mean corpuscular volume; MCH, mean corpuscular hemoglobin; MCHC, mean corpuscular hemoglobin concentration; WBC, total white blood cell count*;

a *(30): For feces consistency score and degree of dehydration*;

b *(32): For rectal temperature, erythrogram, and leukogram–reference values including minimum and maximum 90% CI*.

*Physical examination and hemogram results of Salmonella-negative lactating buffaloes (n = 23, Neg.LB), presented as the minimum (min) and maximum (max) values*.

The health status of the pigs, both *Salmonella*-positive (*n* = 19) and *Salmonella*-negative (*n* = 13) animals, was verified by visual inspection and by feces inspection. In this sense, all animals were asymptomatic (no clinical signs) at the time of swab collection.

## Discussion

### Cross-Species Transmission of *Salmonella* Agona Between Asymptomatic Buffaloes and Pigs

Molecular typing techniques, such us ERIC-PCR and PFGE, are an important method to distinguish different bacterial isolates and are useful to identify the origins of bacteria (33). In our study, *S*. Agona was isolated from two lactating buffaloes, a pregnant sow and a gilt, that generated an identical PFGE pulsetype (Profile 1), revealing a indistinguishable PFGE pattern between these isolates (Table 1 and Figure 1). In this sense, these results bring evidence of a potential cross-species transmission of *S*. Agona between buffaloes and pigs, an important finding, considering that these species can act as asymptomatic reservoirs of *Salmonella* in dairy farms (1–3, 21, 22), contributing for the transmission of this disease to other species and also to humans (1, 3, 21, 22).

Concerning the possible forms of cross-species transmission of *S*. Agona, it is important to consider that buffaloes and pigs of all categories were released in the same paddock during the day, but did not have access to each other's feeders and water system. Therefore, although we have not isolate *S*. Agona from feces inside the paddock, positive isolation of this *Salmonella* serotype from rectal swabs of both species indicate that cross-species transmission likely occurred by fecal-oral route, probably linked to the ingestion of contaminated pasture due to animals shedding *Salmonella* through feces. Also, it was observed in our study that animals did not present clinical signs of Salmonelosis. According to the literature (2), buffaloes infected with *Salmonella* serotypes may be asymptomatic, being that the major source of infection in the herd can be represented by asymptomatic older animals shedding bacteria through feces. In the other hand, a wide spectrum of *Salmonella* serotypes have been associated with a subclinical form of salmonellosis involving asymptomatic healthy pig carriers (21), and thereby these animals are a potential risk factor linked to cross-species transmission, since they can shed bacteria through feces (1, 22, 34).

### *Salmonella* Infection in Asymptomatic Pigs Within the Pig Production Chain

*Salmonella* has been reported worldwide in pigs, with a great diversity of serotypes involved, and it is not uncommon to isolate more than one serotype on the same pig production cycle at a single farm (1, 8, 11–18). In our study, *S*. Agona, *S*. Senftenberg and *S*. Schwarzengrund were the serotypes detected from fecal samples of pigs (Table 1), that did not present diarrhea or other clinical signs of salmonellosis during visual inspection. These serotypes, being *S*. Agona (12, 13, 15, 16, 18), *S*. Senftenberg (18, 35), and *S*. Schwarzengrund (14, 15, 17), have already been isolated in pigs.

*S*. Agona isolates identified from the pregnant sow and the gilt generated an identical PFGE pulsetype (Profile 1), revealing a indistinguishable PFGE pattern between these isolates (Table 1 and Figure 1), while *S*. Agona isolated from the weaned pig generated a different PFGE pulsetype (Profile 2) (Table 1 and Figure 1). Since we sampled each animal at only one timepoint, finding only three animal with a positive result for *S*. Agona could have occurred due to the fact that intermittent shedding of *Salmonella* by pigs is common, and according to literature (36), this can interfere with monitoring and research programs on *Salmonella* infection and the determination of health status in animals. Otherwise, the presence of latent undetectable carriers among infected pigs is a common characteristic in the epidemiology of *Salmonella* (37), and is another fact that must be considered.

*S*. Senftenberg, isolated from four sows, two gilts, two boars, and six weaned pigs, generated an identical PFGE pulsetype (Profile 3), revealing a indistinguishable PFGE pattern between these isolates (Table 1 and Figure 1). In this sense, pig colonization, in our study, could have occurred by horizontal and vertical transmission, or yet by a combination of vertical and horizontal transmission, which is a permanent cycle of contamination on farms (22, 24). It is known that transmission of *Salmonella* between pigs occurs mainly via the fecal-oral route (38) and therefore horizontal transmission between pigs could be occurring, since all animals shared the same installations during the day (paddocks) and during the night (all categories were confined together). Also, it is unlikely that the buffaloes are somehow participating in the transmission of this serotype, since it has not been detected in this specie. In this sense, it is important to consider that, according to literature, serovar Senftenberg has been isolated in pig feed (39), indicating that the consumption of contaminated feed could be the infection source for pigs but not for buffaloes. This can be reinforced by the fact that buffaloes did not have access to pigs feeders and water system.

*S*. Schwarzengrund, isolated from two lactating sows, generated an identical PFGE pulsetype (Profile 4), also revealing a indistinguishable PFGE pattern between these isolates (Table 1 and Figure 1). The fact that *S*. Schwarzengrund was not identified within all the production chain inside the farm, does not mean that this *Salmonella* serotype is restricted to lactating sows. For instance, a study (22) reported that the sensitivity of fecal samples collected on-farm was particularly poor (prevalence of 0%) when comparing to lymph node samples (prevalence of 12.2%) of the pigs at the abattoir. Furthermore, the isolation of *S*. Schwarzengrund in pigs, added to the other two serotypes isolated in pigs in our study, shows the importance of *Salmonella* in this specie and reinforces the theory that a great diversity of serotypes can be involved in the infection cycle inside a single farm.

The results of this work confirmed a potential transmission of *S*. Agona between buffaloes and pigs. Also, evidenced a potential *Salmonella* infection within the pig production phases. Therefore, considering the epidemiological importance of buffaloes and pigs in the cycle of *Salmonella* infection, the results presented reinforce the importance of the correct establishment of preventive health strategies in farms, in special the importance of avoiding contact between buffaloes and pigs when raised in the same farm, since cross-species transmission can occur, increasing the risk of spreading the disease.

## Data Availability Statement

The original contributions presented in the study are included in the article/supplementary material, further inquiries can be directed to the corresponding author/s.

## Ethics Statement

The animal study was reviewed and approved by The Ethics Committee on Animal Use of Faculdade de Ciências Agrárias e Veterinárias, UNESP (Protocol no 010885-08). Written informed consent for participation was not obtained from the owners because at the time of the execution of the project, the written consent was not a requirement of the Ethics Committee on Animal Use of “Faculdade de Ciências Agrárias e Veterinárias, UNESP”. Therefore, only a verbal consent was given.

## Author Contributions

AS was responsible for the conception of the study, experimental design, collection of samples in the farm, part of laboratory work, data analysis and interpretation, and manuscript writing. DS and JF were responsible for the conception of the study, experimental design, part of laboratory work, data analysis and interpretation, and manuscript revision. RM was responsible for the conception of the study and part of the laboratory work. LP and KS were responsible for the collection of samples on the farm. CS and DR were responsible for part of the laboratory work. SR was responsible for data analysis and interpretation and manuscript revision. All authors contributed to the article and approved the submitted version.

## Conflict of Interest

The authors declare that the research was conducted in the absence of any commercial or financial relationships that could be construed as a potential conflict of interest.
